# Design of a Low-Noise 2.4/5.5 GHz Dual-Band LNA Based on Microstrip Structure

**DOI:** 10.3390/mi17010018

**Published:** 2025-12-24

**Authors:** Mingwen Zhang, Zhiqun Cheng, Tingwei Gong, Bangjie Zheng, Zhiwei Zhang

**Affiliations:** 1 School of Electronics and Information, Hangzhou Dianzi University, Hangzhou 310018, China; mwzhang@wuyiu.edu.cn (M.Z.);; 2 School of Mechanical and Electrical Engineering, Wuyi University, Wuyishan 354300, China; 3 The Key Laboratory for Agricultural Machinery Intelligent Control and Manufacturing of Fujian Education Institutions, Wuyishan 354300, China; 4 The Higher Educational Engineering Research Center for Digital and Intelligent Development of the Whole Bamboo Industry Chain of Fujian Province, Wuyishan 354300, China; 5 School of Information Engineering, Xinjiang Institute of Technology, Akesu 843100, China

**Keywords:** transmission line, radial stub, dual-band LNA, dual-band bias circuit

## Abstract

This paper presents a 2.4/5.5 GHz single-stage dual-band low-noise amplifier (DB-LNA) based on a microstrip structure. The design utilizes a purely microstrip dual-band bias circuit (DBBC), composed of series microstrip lines and radial stubs. The broadband characteristics of the radial stubs enable wide frequency coverage and good frequency selectivity. A simple series-shunt microstrip matching network is adopted to maintain a compact overall design structure. The proposed DB-LNA is fabricated using a standard printed circuit board (PCB) process. Measurement results show that the amplifier achieves gains of 15.6 dB and 12.3 dB, input return losses of 14.6 dB and 14.5 dB, and output return losses of 23.2 dB and 14.1 dB at 2.4 GHz and 5.5 GHz, respectively. The measured noise figures (NF) are 1.0 dB and 1.1 dB at the corresponding frequencies, with −3 dB bandwidths exceeding 200 MHz. Compared with existing designs, the proposed LNA demonstrates notable advantages in both noise performance and bandwidth, while occupying a compact area of only 75 × 43 mm^2^.

## 1. Introduction

With the rapid evolution of modern communication networks toward higher speeds and greater intelligence, there is an urgent industry demand for WLAN communication systems that offer strong compatibility, compact size, and support for multi-frequency bands. As a key module in the receiver front-end, LNAs are also required to simultaneously adapt to multiple operating frequency bands and communication standards. However, traditional multi-band LNAs often adopt wideband designs [1] or switching architectures [2]. While the former covers a broad frequency range, it is prone to introducing out-of-band interference signals, leading to channel congestion. The latter enables band switching but can only operate in a single frequency band at a time, and the switching components introduce parasitic parameters that degrade system performance. In contrast, LNAs based on a concurrent dual-band structure [3,4,5,6,7,8,9,10,11,12] strike a favorable balance between bandwidth utilization and design complexity, making them a focus of increasing research attention.

Traditional DB-LNAs often face both process–structure challenges in implementation. While solutions based on CMOS [2] or GaAs [1,4,9] technologies offer mature performance, they inherently suffer from limitations such as complex layout design, high fabrication costs, and lengthy manufacturing cycles. In contrast, microstrip structures have emerged as a viable alternative due to their design flexibility and cost advantages. However, conventional microstrip-based DB-LNAs typically rely heavily on discrete components (e.g., capacitors, resistors, and inductors) to construct biasing circuits, matching networks, RF chokes, and DC blocking/bypass circuits. This not only introduces component tolerances and additional noise but also leads to performance deviations from design expectations due to soldering uncertainties, thereby compromising circuit consistency and reliability. Several studies have attempted to reduce the number of discrete components, though each approach has certain drawbacks. For example, Ref. [3] adopted the SISL technology to realize a bias and matching network without discrete components, achieving NF of 0.7 dB and 1.1 dB at 2.45/5.25 GHz dual bands, respectively. However, this process is relatively complex. Ref. [5] employed resistors, inductors, and capacitors to form a wideband bias circuit with a simple matching structure, but still required external capacitors, leading to increased NF of 1.5/1.6 dB. Refs. [6,7] proposed microstrip bias structures that still incorporated passive discrete resistors in series, resulting in an NF exceeding 2.85 dB. Ref. [8] utilized PCB technology, replacing inductors with parallel-coupled lines and incorporating discrete components to realize a compact 2.4/5.5 GHz LNA, yet the NF remained as high as 1.6 dB. Ref. [9] demonstrated the good performance of a microstrip bias circuit in a tri-band power amplifier (PA), providing a useful reference for LNA design. A synthesis of existing results indicates that to improve the noise performance and precision of microstrip-based LNAs, it is essential to minimize or even completely eliminate the use of discrete components in bias and matching circuits. Furthermore, conventional bias networks often employ large-value choke inductors, which are prone to self-resonance limitations and are mostly suitable for hybrid circuits or low-frequency applications [10]. Therefore, there is an urgent need in current microstrip-based DB-LNA design to develop pure microstrip-structured DBBC and matching networks that can simultaneously meet multi-band operation requirements and low-noise performance.

This paper proposes a pure microstrip bias circuit implemented with series microstrip lines and radial stubs, eliminating the need for soldered discrete components. The designed circuit supports concurrent dual-band operation with a relatively wide usable bandwidth, thereby reducing the complexity of the matching network design. For the input matching network (IMN), a broadband structure consisting of series microstrip lines and open-circuited stubs is employed to achieve impedance matching while satisfying low-noise requirements. The output matching network (OMN) is accomplished using only series microstrip lines to provide conjugate matching and optimize gain performance. Section 2 details the design methodology of the pure microstrip DBBC and matching networks. Section 3 presents the simulated and measured results of the DB-LNA based on the proposed technique, along with corresponding analysis and conclusions.

## 2. Design Theory and Analysis

### 2.1. Topology of the DB-LNA

**Figure 1:**
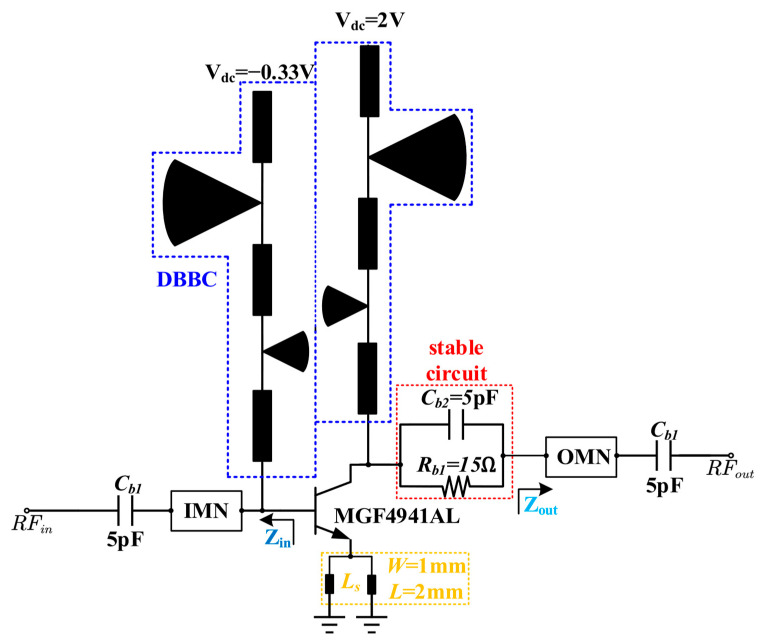
The schematic diagram of the DB-LNA.

This paper presents the design of a single-transistor DB-LNA, as illustrated in Figure 1. To circumvent parasitic effects introduced by discrete components and solder joints in traditional designs, a purely microstrip-based structure is adopted to implement both the DBBC and the matching networks. The DBBC not only provides a stable operating point for the transistor but also exhibits high impedance at the two target frequency bands, effectively preventing RF signal leakage and selecting the desired operating bands. Regarding the matching design, the IMN is designed to match the gate impedance to 50 Ω for optimized noise performance, while the OMN conjugately matches the drain impedance to 50 Ω to maximize gain. Throughout the design process, a source degeneration inductor *L_s_* is utilized to bring the maximum gain circles and minimum noise circles closer together, thereby simplifying the matching structure while maintaining performance. To further ensure stability, a stabilization circuit consisting of a parallel combination of *R_b_*_1_ and *C_b_*_2_ is incorporated between the transistor drain and the output matching network.

### 2.2. DBBC Design

Conventional DBBCs are often implemented using passive discrete components, such as large resistors shunted with bypass capacitors [1,8] or large inductors shunted with bypass capacitors [10]. However, these components introduce additional noise, and their ultra-wideband characteristics can allow out-of-band noise to enter the system, degrading the overall noise performance. As the operating frequency of DB-LNAs increases, these effects become more pronounced. Consequently, Ref. [5] shows that their NF are typically above 1.5 dB. Another approach utilizes distributed elements, employing quarter-wavelength microstrip lines combined with bypass capacitors [9]. While this method avoids the additional noise introduced by discrete components, its operating bandwidth remains relatively wide, potentially admitting interference from unwanted frequency bands. Furthermore, it becomes challenging to achieve satisfactory performance at both bands when the frequency separation is large [13,14,15]. In contrast, studies [16] indicate that a T-shaped microstrip structure can emulate quarter -wavelength lines at two distinct frequencies. This configuration offers advantages such as flexible parameter adjustment, good dual-band matching performance, and relatively wide bandwidth, making it more suitable for DBBC applications.

**Figure 2:**
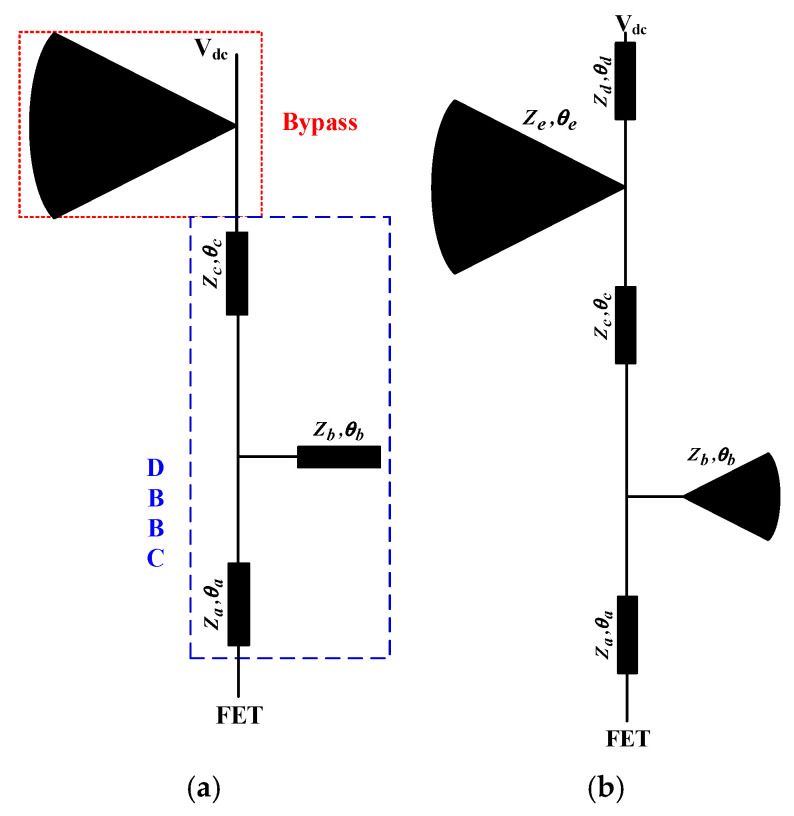
(**a**) Conventional DBBC structure. (**b**) The proposed improved DBBC structure.

Figure 2a shows a conventional T-shaped DBBC, consisting of two series microstrip lines (*Z_a_*, *θ_a_* and *Z_c_*, *θ_c_*) and one parallel open stub (*Z_b_*, *θ_b_*). The series line (*Z_a_*, *θ_a_*) and the parallel stub (*Z_b_*, *θ_b_*) work together to create a high impedance at the higher frequency, with (*Z_a_*, *θ_a_*) typically being a quarter-wavelength at this frequency. The series line (*Z_c_*, *θ_c_*) is responsible for providing high impedance at the lower frequency. However, constrained by the physical dimensions of (*Z_c_*, *θ_c_*), this structure often results in a narrow bandwidth or struggles to achieve the desired performance when the frequency separation is significant. In the traditional T-structure, the radial microstrip stub primarily acts as a bypass capacitor and can sometimes be replaced by a discrete capacitor. To achieve wider frequency coverage at the target bands, this paper, referring to [17,18], adopts a purely microstrip-based DBBC structure evolved from the traditional T-type configuration. This circuit eliminates the need for any discrete passive components. As illustrated in Figure 2b, a short radial microstrip stub (*Z_b_*, *θ_b_*) is used to replace the original parallel open stub. In this design, (*Z_a_*, *θ_a_*) and (*Z_b_*, *θ_b_*) are still responsible for the high-impedance characteristic in the high-frequency band, while the entire structure cooperatively to achieve high impedance in the low-frequency band. The radial stub (*Z_e_*, *θ_e_*) here serves not only a bypass function but also participates in impedance matching. Benefiting from the inherent broadband characteristics of the radial stub and the flexibility provided by the multi-section microstrip line combination, this structure can achieve high impedance at the target frequencies under various parameter combinations, thereby effectively extending the operating bandwidth.

**Figure 3:**
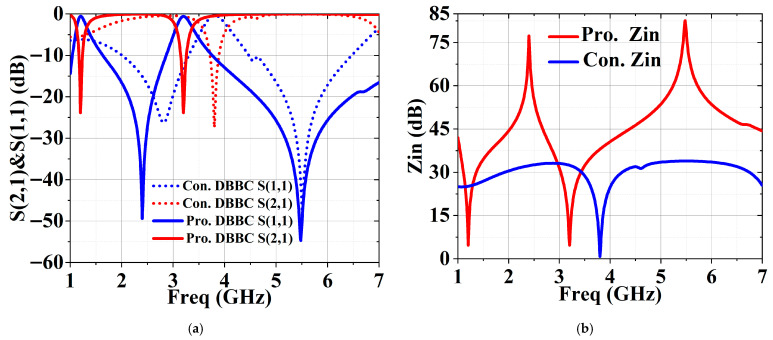
(**a**) S (1,1) and S (2,1) of the DBBC. (**b**) Input impedance of the DBBC.

To validate the performance of the proposed DBBC, Figure 3a comparatively presents the frequency coverage of the conventional DBBC in Figure 2 and the proposed DBBC, defined by S (2,1) < −20 dB. The proposed circuit achieves a low-frequency band from 2.3 to 2.5 GHz (200 MHz bandwidth) and a high-frequency band from 4.9 to 6.1 GHz (1200 MHz bandwidth), which significantly exceeds the results of the traditional structure and those reported in [5,6,7,8]. As shown in Figure 3b, the proposed DBBC achieves a higher input impedance than the conventional one, which is a key requirement for effective RF signal blocking.

### 2.3. Matching Circuit Design

**Figure 4:**
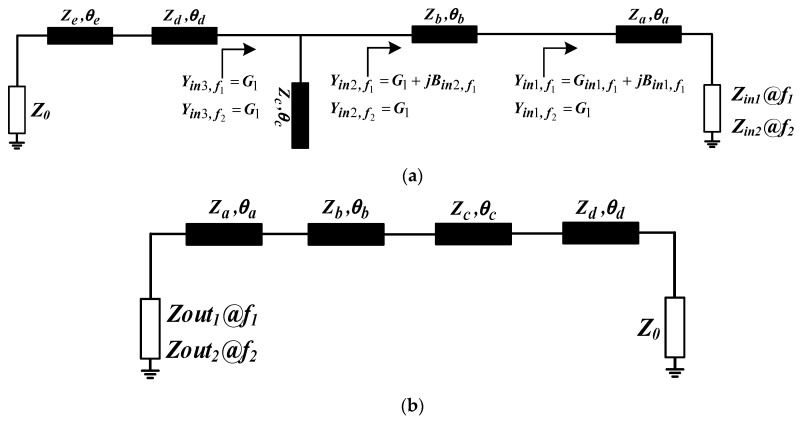
(**a**) Schematic for the IMN. (**b**) Schematic for the OMN.

The matching circuit plays a critical role in LNA design. Once the bias point is determined, the overall operating characteristics of the circuit are fixed. However, limited by PCB processes, the real part of the resistance looking into the transistor’s gate and drain is typically small, resulting in a significant separation between the maximum gain circle and the minimum NF circle, a challenge particularly pronounced in dual-band designs. Furthermore, LNA stability is often insufficient. To address this, a source degeneration inductor *L_s_* is commonly introduced, forming series negative feedback to bring *Z_opt_* (the optimum noise impedance) closer to *Z_ms_* (the impedance for maximum gain) while improving stability. Nevertheless, relying solely on source degeneration is usually inadequate to meet the design requirements. Therefore, the IMN must be designed to achieve *Z_in_* = *Z*_0_ (equal-value matching) to minimize noise, while the OMN should provide Z_out_* = *Z*_0_ (conjugate matching) to maximize gain. Figure 4a illustrates the IMN structure adopted in this work and its matching process. The structure consists of series microstrip lines and open stubs. The input impedance *Z_in_* looking into the transistor gate at two arbitrary frequencies *f*_1_ and *f*_2_ can be expressed as unequal complex impedances: *Z_in_*_1_ = *R*_1_ + *jX*_1_ and *Z_in_*_2_ = *R*_2_ + *jX*_2_. Through transmission line segments a and b, these two complex impedances are transformed into admittances with equal real parts, where one becomes a pure conductance and the other remains a complex admittance. Subsequently, the shunt stub c is used to cancel the susceptance component of the complex admittance. Finally, with the aid of transmission line segments d and e, the two equal real resistances are matched to the system characteristic impedance *Z*_0_ at both frequencies.

(1) $$R_{in1,f_{2}}(Z_{a}-X_{2}\tan\theta_{a,f_{2}})-R_{2}Z_{a}=0$$

(2) $$Z_{a}(X_{2}+Z_{a}\tan\theta_{a,f_{2}})-R_{in1,f_{2}}R_{2}\tan\theta_{a,f_{2}}=0$$

Applying traditional transmission line theory yields the following equations:

Substituting Equation (1) into (2) and rearranging through some straightforward manipulation gives:

(3) $$\theta_{a,f_{2}}=\arctan\left(\frac{\Delta \pm \sqrt{\Delta \Delta^{2}+4X_{2}^{2}Z_{a}^{2}}}{2X_{2}Z_{a}}\right)+n\pi$$

(4) $$\frac{1}{G_{1}}=R_{1}=R_{in1,f_{2}}=\frac{R_{2}Z_{a}}{Z_{a}-X_{2}\tan\theta_{a,f_{2}}}$$

Here, $\Delta =Z_{a}^{2}-R_{2}^{2}-X_{2}^{2}$. Subsequently, considering that the shunt stub c is connected in parallel, the design is analyzed using the admittance method.

(5) $$G_{1}(Y_{b}-B_{in1,f_{1}}\tan\theta_{b,f_{1}})-G_{in1,f_{1}}(Y_{b}+B_{in2,f_{1}}\tan\theta_{b,f_{1}})=0$$

(6) $$B_{in2,f_{1}}(Y_{b}-B_{in1,f_{1}}\tan\theta_{b,f_{1}})-Y_{b}(B_{in1,f_{1}}+Y_{b}\tan\theta_{b,f_{1}})+G_{1}G_{in1,f_{1}}\tan\theta_{b,f_{1}}=0$$

With *Y_b_*, *G_in_*_1_, *f*_1_, *B_in_*_1_, *f*_1_, and *G*_1_ given in Equations (5) and (6), one can solve for *θ_b_* from Equation (5).

(7) $$B_{in2,f_{1}}=\pm \sqrt{\frac{G_{1}(G_{1}^{2}-B_{in1,f_{1}}^{2}+G_{in1,f_{1}}^{2}-2G_{1}G_{in1,f_{1}})}{G_{in1,f_{1}}}}$$

Then, the value of the other unknown, *θ_b_*, *f*_1_, is readily found.

(8) $$\theta_{b,f_{1}}=\arctan\left(G_{1}\frac{G_{1}-G_{in1,f_{1}}}{G_{1}B_{in1,f_{1}}+B_{in2,f_{1}}G_{in1,f_{1}}}\right)+n\pi$$

Fortunately, the shunt stub c does not disturb the match at *f*_2_, where *Z_in3_*, *f*_2_ = *R*_1_ is maintained, requiring *Y_Stub_*_,*f*2_ = 0. At *f*_1_, however, its input admittance must equal −*B_in_*_2,*f*1_, as given by:

(9) $$Z_{c}=\cot(2m\pi )/B_{in2,f_{1}}$$

The parameters of microstrip segments d and e are determined following the method in [8]. Likewise, the OMN uses the configuration illustrated in Figure 4b, and its analysis is analogous to the one described above.

**Figure 5:**
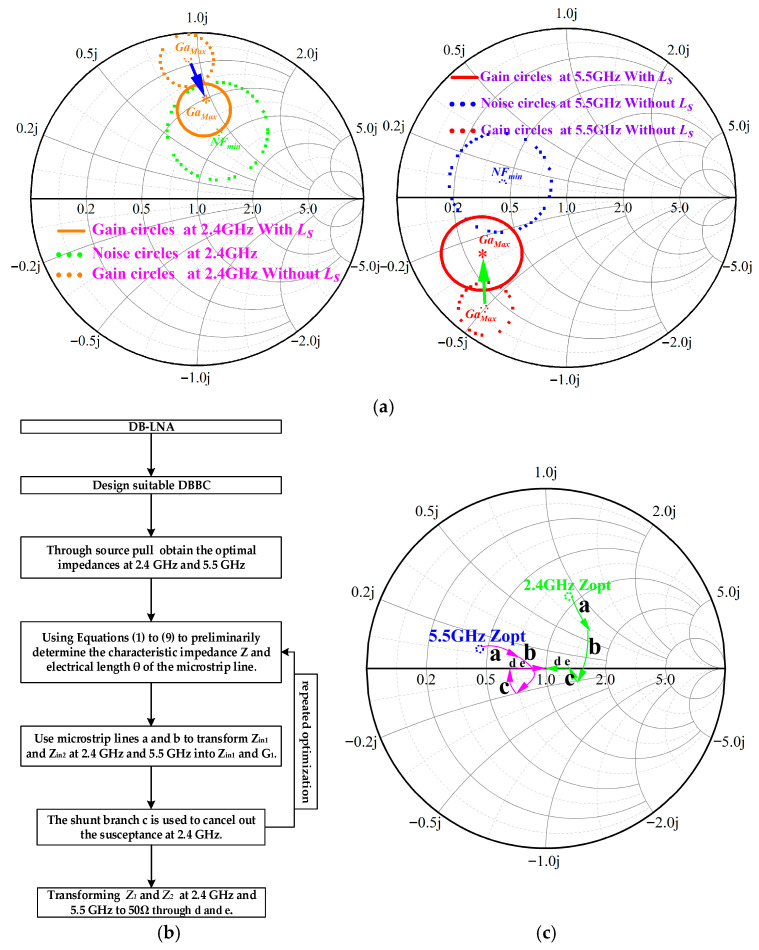
(**a**) Main process flowchart of the proposed method. (**b**) A comparison of the positions of the maximum gain and minimum noise figure circles with and without Ls. (**c**) A schematic of the input matching process.

The aforementioned method is summarized in the flowchart of Figure 5a. Figure 5b illustrates the shifts in the available gain circles and NF circles at 2.4 GHz and 5.5 GHz, with and without the *Ls*. It can be observed that after introducing *Ls*, the NF circles remain largely unchanged, while the available gain circles move closer to the NF circles, thereby facilitating a better gain-noise trade-off. Figure 5c further demonstrates the specific matching process of the IMN.

## 3. Implementation and Measurement

**Figure 6:**
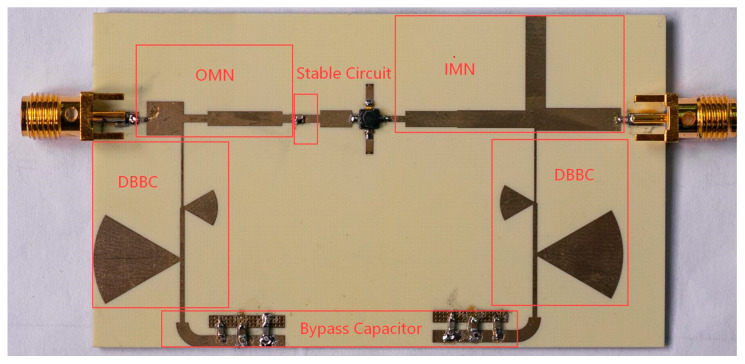
The fabricated DB-LNA.

Based on the theoretical analysis above, a 2.4/5.5 GHz DB-LNA has been fabricated using the MGF4941AL transistor (Mitsubishi Electric, Tokyo, Japan). The design incorporates the DBBC and matching networks analyzed in Section 2 as the biasing, IMN and OMN structures. The circuit is fabricated on a Rogers 4350B substrate (Rogers Corporation, Chandler, AZ, USA) with a thickness of 0.762 mm, a dielectric constant of 3.66, and a loss tangent of 0.0037. Passive components, including resistors and capacitors, are selected from Panasonic’s ERJ2GEJ series (Panasonic, Shenzhen China) and Murata’s GJM1555C1H series (Murata Electronics, Jiangsu, China), respectively. A photograph of the fabricated PCB, which measures 75 mm × 43 mm, is shown in Figure 6, with key components such as the DBBC, IMN, and OMN clearly labeled.

**Figure 7:**
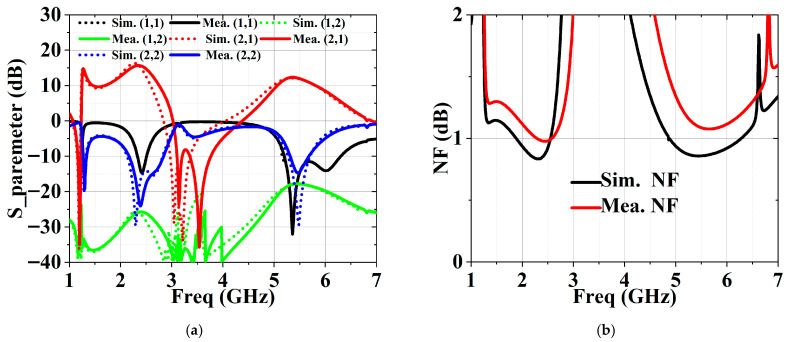

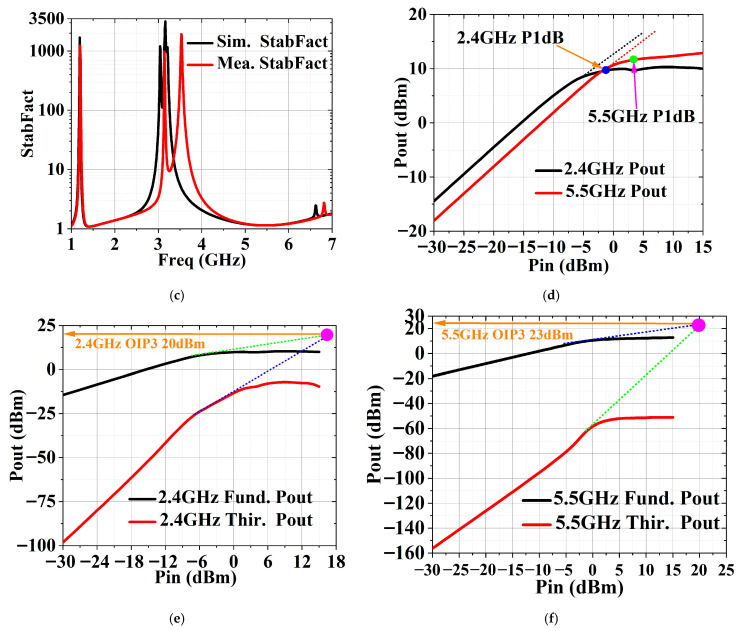
Simulated and measured performance: (**a**) S-parameter, (**b**) NF, (**c**) StabFact, (**d**) P1dB, (**e**) 2.4 GHz OIP3, (**f**) 5.5 GHz OIP3.

The S-parameters of the fabricated DB-LNA were measured using a Siglent SNA5000A vector network analyzer (Siglent, Shenzhen China), while the noise performance was characterized with a Siglent SSA5000A spectrum analyzer (Siglent, Shenzhen China). Linearity was evaluated using an N5244A vector network analyzer (Keysight Technologies, Inc., Santa Rosa, CA, USA) combined with a U2021XA power sensor (Keysight Technologies, Inc., Santa Rosa, CA, USA). Simulated and measured results are presented in Figure 7, demonstrating that the LNA covers frequency ranges of 2.3–2.5 GHz and 5.2–5.6 GHz. Within these bands, the measured S_11_ values are −14.6 dB and −14.5 dB, S_22_ values are −23.2 dB and −14.1 dB, S_21_ values are 15.6 dB and 12.3 dB, and the NF are 1 dB and 1.1 dB. The stability factor remains greater than 1 across the operating bands. Linearity was evaluated by applying a single-tone signal with RF power swept from −30 dBm to 15 dBm. The resulting P1dB and OIP3 at 2.4 GHz and 5.5 GHz are shown in Figure 7d–f, respectively. Owing to the DBBC configuration, the proposed LNA exhibits excellent linearity across the design bands.

Table 1 presents a performance comparison between the proposed DB-LNA in this work and those reported in the literature. As the table shows, the proposed DB-LNA demonstrates significant advantages in terms of NF, power consumption, and frequency band coverage.

## 4. Conclusions

This paper presents a low-cost, microstrip-based DB-LNA. The design employs a DBBC combining radial stubs and series microstrip lines, while the matching network also adopts a purely microstrip-based topology. Measurement results demonstrate excellent performance during dual-band operation at 2.4/5.5 GHz, with a NF close to 1 dB. The proposed DB-LNA shows strong potential for WLAN applications.

## Figures and Tables

**Table 1:** Performance comparison of DB-LNAs.

Ref.	Frequency (GHz)	S11 (dB)	S22 (dB)	S21 (dB)	NF (dB)	Size (mm^2^)	Power (mW)	Technology
[4]	2.4	−11.3	−24.6	33.84	0.946	-	-	GaAs MMIC
	5.75	−17.4	−11.1	20	0.493			
[5]	2.45	−20	-	22	1.5	30 × 30	7.5	HMIC
	5.2	−21	-	12	1.6			
[6]	2.3–2.5	−8.5	-	3–12.2	0.5–5	55 × 60	41.25	HMIC
	4.2–4.6	−15	-	9.5–12.9	2.5–5			
[7]	2.44	−10.5	-	7.15	4.34	-	35.1	HMIC
	5.25	−15.9	-	7.8	4.69			
[8]	2.33–2.46	−29.8	−15.2	20.3	1.6	16 × 85	39.3	HMIC
	5.43–5.58	−20.3	−16.4	14.7	1.6			
[10]	2.4	−25	-	11.6	3.96	120 × 34	56	HMIC
	5.7	−12	-	8.9	2.89			
This Work	2.3–2.5	−14.6	−23.2	15.6	1	75 × 43	20	HMIC
	5.2–5.6	−14.5	−14.1	12.3	1.1			

## Data Availability

The data presented in this work are available within the article.
